# Current Options and Future Directions in Immune Therapy for Glioblastoma

**DOI:** 10.3389/fonc.2018.00578

**Published:** 2018-12-05

**Authors:** John Lynes, Victoria Sanchez, Gifty Dominah, Anthony Nwankwo, Edjah Nduom

**Affiliations:** ^1^National Institute of Neurological Disorders and Stroke, Bethesda, MD, United States; ^2^MedStar Georgetown University Hospital, Washington, DC, United States

**Keywords:** glioblastoma, immunotherapy, virus, vaccination, checkpoint, sequencing, cell therapy

## Abstract

Glioblastoma is in need of innovative treatment approaches. Immune therapy for cancer refers to the use of the body's immune system to target malignant cells in the body. Such immune therapeutics have recently been very successful in treating a diverse group of cancerous lesions. As a result, many new immune therapies have gained Food and Drug Administration approval for the treatment of cancer, and there has been an explosion in the study of immune therapeutics for cancer treatment over the past few years. However, the immune suppression of glioblastoma and the unique immune microenvironment of the brain make immune therapeutics more challenging to apply to the brain than to other systemic cancers. Here, we discuss the existing barriers to successful immune therapy for glioblastoma and the ongoing development of immune therapeutics. We will discuss the discovery and classification of immune suppressive factors in the glioblastoma microenvironment; the development of vaccine-based therapies; the use of convection-enhanced delivery to introduce tumoricidal viruses into the tumor microenvironment, leading to secondary immune responses; the emerging use of adoptive cell therapy in the treatment of glioblastoma; and future frontiers, such as the use of cerebral microdialysis for immune monitoring and the use of sequencing to develop patient-specific therapeutics. Armed with a better understanding of the challenges inherent in immune therapy for glioblastoma, we may soon see more successes in immune-based clinical trials for this deadly disease.

## Introduction

GBM is an often-fatal brain malignancy that accounts for the majority of primary malignant brain tumors (1, 2) and has a recurrence rate of more than 90% (3). The current standard treatment for patients with GBM is maximal safe resection of the tumor followed by radiotherapy with temozolomide (TMZ), but survival is poor, with a median survival of just over 14 months (4). While other treatments, such as Gliadel wafers (5), bevacizumab (6), and tumor treatment fields (7), have been cleared by the Federal Drug Administration (FDA) for the treatment of glioblastoma, no other treatment has been accepted by the neuro-oncology community as standard of care, due to the inability of these treatments to significantly affect overall survival.

Immunotherapy is a rising field of study wherein one's own immune system is manipulated to target cancer antigens. Though the first report of a connection between tumor regression and infection was by Chekhov in 1884 (8), the concept of immune therapy for cancer is often attributed to the first use of Coley's toxin in 1893. William Coley, a “bone surgeon,” inoculated sarcoma patients with heat-inactivated *streptococcus* after observing a case of a patient having tumor regression after accidental infection (9). Over a century later, there have been several breakthroughs in the field of immune-oncology, leading to the FDA approval of several new agents, including checkpoint inhibitors.

Checkpoint inhibitors nivolumab, an anti-programmed death-1 (PD-1) antibody, and ipilimumab, an anti-cytotoxic T-lymphocyte-associated protein 4 (CTLA-4) antibody, demonstrated increased survival in untreated melanoma (10) and were FDA approved in 2015. Pembrolizumab, another anti-PD-1 antibody, has shown benefit in non-small cell lung cancer (11) and was FDA approved in 2017. Chimeric Antigen Receptor (CAR) T-cell therapy and blinatumomab, a targeted antibody against CD19, were approved for pediatric leukemias in 2017. In parallel with these advances, numerous groups have pursued strategies for immunotherapy in glioblastoma, given its recalcitrance in the face of traditional therapies. However, glioblastoma has remained a challenging disease to treat with immune therapeutics, as it has been a challenge with conventional therapeutics.

It was previously believed that the brain was immune privileged (12), because it could not induce an adequate immune response in the case of graft rejection. This led to understandable skepticism regarding the use of immune therapy for these lesions. However, new insight has revealed that the CNS, in communication with the rest of the body, can mount appropriate immune responses (13). Despite this, the success of immune therapy is not guaranteed. Immune therapy for glioblastoma is limited by the immunosuppressive mechanisms in the glioblastoma microenvironment (14). Therefore, scientists are working to determine the role that these different immunosuppressive factors play in tumor formation and progression.

This review aims to highlight the development of immune therapy for primary brain malignancies. Specifically, we will provide a detailed review of key players of immune suppression in the tumor microenvironment and outline the development of new immune treatments for glioblastoma. These new immune therapeutics include: checkpoint inhibition, tumor vaccines, adoptive cell therapies and convection enhanced delivery of tumoricidal viruses. Finally, we will discuss areas of future research for immune therapy, including advances in immune biomarker development.

## Immunophenotyping the Tumor Microenvironment

**Figure 1:**
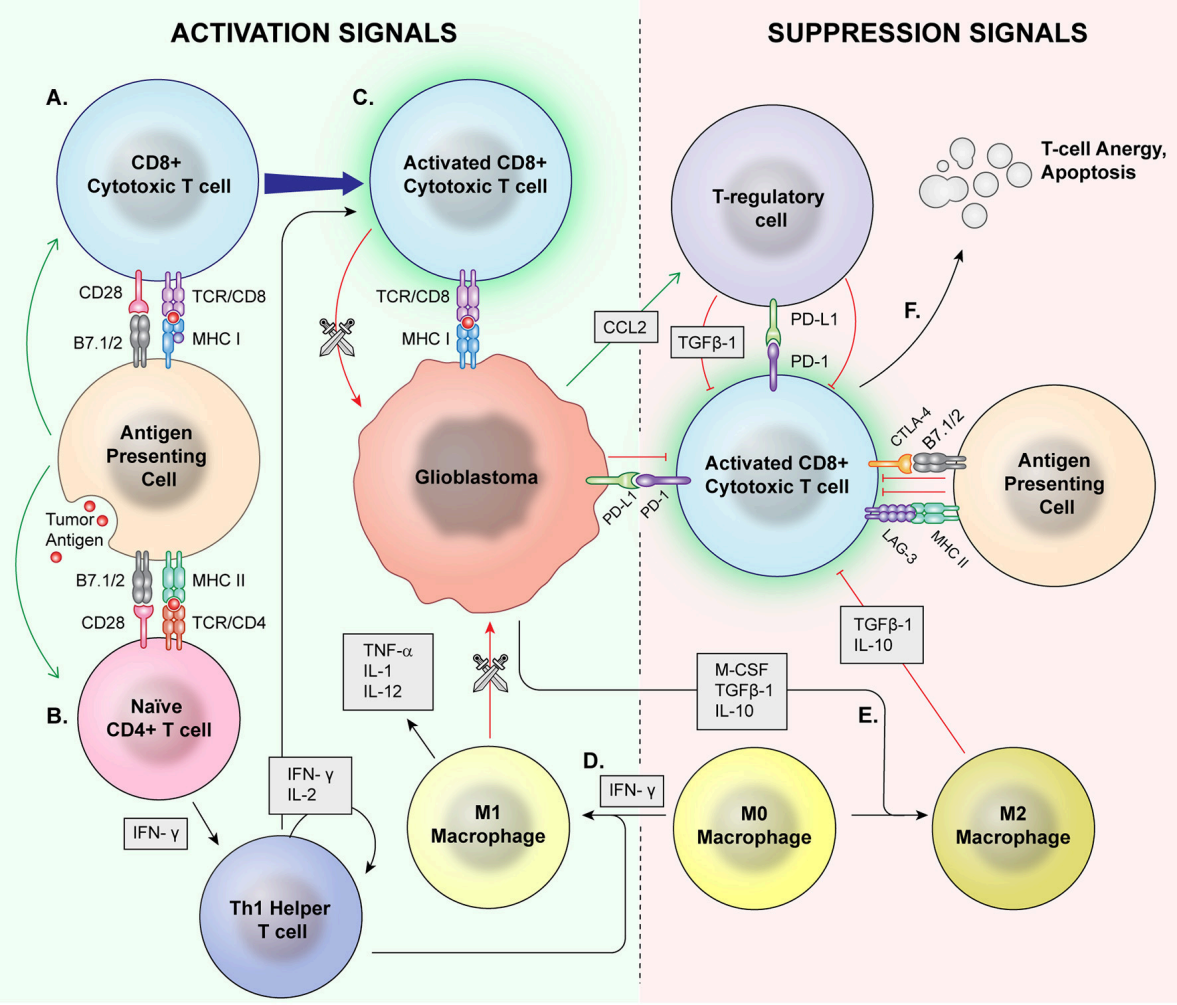
Normal Inflammation vs. Immunosuppression Mechanisms. Antigen presenting cells (APCs) phagocytose tumor antigens and present to cytotoxic T cells as well as naïve CD4+ cells. Via coactivation signals, the APCS activate the cytotoxic T cells **(A)** and skew helper T cells to a proinflammatory Th1 lineage **(B)**. The activated cytotoxic T cells then recognize and attack malignant cells **(C)**. T regulatory cells, M2 macrophages, and MDSCs are major mediators of immune suppression. M0 macrophages may be skewed toward a pro-inflammatory M1 phenotype by IFN-γ **(D)**, which directly phagocytose target cells and release proinflammatory cytokines. **(E)** Glioblastoma cells also signal M0 macrophages to skew toward an M2 phenotype which release immunosuppressive cytokines. Immune checkpoints induce anergy and apoptosis of CD8+ cytotoxic T cells **(F)** and CD4+ cells.

Immunophenotyping, or the description of the immune system's form and functioning in the tumor microenvironment, has emerged as an important factor in understanding tumorigenesis, tumor survival, and potential for utilizing the immune system against glioblastoma. A variety of immune cell types are found in this environment with complex, still incompletely understood interactions (Figure 1).

### Regulatory T Cells

Several cell types have been associated with the immunosuppressive glioblastoma microenvironment. Regulatory T Cells (Tregs), traditionally CD4+CD25+ FoxP3+ lymphocytes, help balance the immune system in a non-pathologic context, preventing injury from excessive activity and autoimmune disease (15). These cells induce a shift toward the T-Helper-2 (TH2) immune phenotype and immunosuppressive cytokine production. However, Tregs are found in the blood of glioblastoma patients at a higher ratio to CD4+ non-Tregs as compared to healthy controls (16). Glioblastoma cells have been found to release chemokines that attract Tregs to the tumor microenvironment (17) via the chemokine CCL2 (18). Most Tregs found in the tumor microenvironment are naturally occurring Tregs of thymic origin rather than induced Tregs (19). While CD4+ Tregs have been under extensive investigation for decades, the existence and role of CD8+ Tregs is less well studied and is not well understood in glioblastoma. First described by Damle in the 1980s, CD8+ Tregs are CD8+ T cells that are immunosuppressive (20), similar to CD4+ Tregs Kiniwa et al. would go on to describe CD8+ Tregs from an oncologic perspective in prostate cancer (21). Subsequent groups would describe these immunosuppressive cells in colorectal cancer (22), hepatocellular carcinoma (23), non-small cell lung cancer (24), and ovarian cancer (25). However, similar studies have not yet been performed in glioblastoma.

### Macrophages and Myeloid Derived Suppressor Cells

Monocyte-derived macrophages or microglia native to the CNS can constitute as high as 12% of glioblastoma mass (26) and have been associated with poor outcome in non-CNS malignancies (27, 28). These tumor-associated macrophages can be nonpolarized M0 macrophages, classical (M1) lineage or M2 lineage. M1 macrophages upregulate cell surface molecules associated with antigen presentation and recognition; release proinflammatory cytokines such as TNF-α, IL-1β, and IL-12; and directly phagocytose targets (29). The M2 macrophages, however, help mediate immunosuppression and tumor invasion (30). Studies have suggested that the M2 lineage has been found in disproportionately high concentration in the glioblastoma environment (31) and their prevalence has been associated with glioma grade (32). This M2 immunosuppressive state is maintained by multiple signals, including the TGF-beta pathway and TH2 cytokines IL-4 and IL-10 (33). Conversely, interferon-gamma activates the inflammatory M1 phenotype (34). RNA sequencing of tumor associated macrophages in murine models and human samples suggests that a majority of macrophages found in glioblastoma are bone-marrow derived, rather than resident microglia, and that they have enriched proliferation and migration gene expression (35, 36). Recently, whole-genome analysis and microRNA expression profiling performed on human tumor tissue suggested that the macrophage population represents a spectrum rather than strict lineages, with the largest share most similar to M0 macrophages (37). Driving differentiation of TAMs to an M1 phenotype could be a new treatment approach. MiR-146, a microRNA found to direct hematopoietic differentiation (38), was found to be downregulated in glioblastoma macrophages and may direct the M0 phenotype toward a pro-inflammatory M1 lineage. Additionally, miR-142-3p is downregulated in glioma TAMs, and administration of miR-142-3p in a murine glioma model decreased infiltrating TAMs and extended median survival (39). A shift toward an M1 phenotype has been associated with response to therapy in a murine glioma model (40). However, iatrogenic sources may also affect balance of M1 and M2 macrophages, with one study finding that radiation therapy increased the proportion of M2 macrophages in *in-vivo* murine glioma models due to increased radiosensitivity of the M1 line (41).

Myeloid derived suppressor cells (MDSCs), precursors to both macrophage lineages, are found in higher levels in the serum of glioblastoma (42). This cell type is generally considered to have an overall immunosuppressive phenotype, though there have been reports of antitumor effects via nitrous oxide release (43) and potential M1 macrophage characteristics (44). However, in glioblastoma, these cells are immunosuppressive (45). MDSCs promote Treg proliferation (46), create oxidative stress that inhibits T cell proliferation (47), and deplete L-arginine, inhibiting CD3 production and T cell proliferation (48). Gielen et al describe a trend toward increased circulating MDSCs in glioma patients dependent on tumor grade, as well as increased arginase activity of these MDSCs compared to healthy controls (49). Additionally, immature monocytes have been found to express CTLA-4 (50) and PD-L1 (51), prominent immune checkpoints, which are membrane proteins associated with modulating T cell activation and anergy. Targeting MDSCs has shown benefit in pre-clinical models of non-CNS tumors (52), and a phase I trial is currently underway to evaluate if this strategy may be effective in glioblastoma (53).

### Immune Checkpoints

In the physiologic state, immune checkpoints play an important role in the constant balance of immune modulation in order to prevent autoimmunity (54). In typical activation of T cells, coactivation of two receptor-ligand pairs is required. Major histocompatibility complex (MHC) I and II are key components of antigen presentation and recognition that make up one half of this co-stimulatory pathway. CD28/B7 interaction often acts as the second signal. Antigen presenting cells (APCs) activate cytotoxic T cells and T helper cells by presenting a tumor antigen on the MHC complex and providing co-stimulation (55). Cytotoxic T cells directly attack tumor cells that express a targeted antigen while helper T cells propagate an antitumor immune response via release of proinflammatory cytokines and induction of immune memory B and T cells (56). Immune checkpoints are regulators of the immune system that are expressed by T effector cells, APCs, and myeloid-derived cells in the normal immune system. When these checkpoints are engaged, they decrease immune activity by promoting T-cell anergy and apoptosis (57), preventing T-cell co-stimulation and activation by antigen presenting cells (58), and promoting Treg suppression of T effector cell functioning (59), among other mechanisms of immunosuppression. However, in the tumor microenvironment, tumor cells may utilize these pathways to suppress an effective immune response targeted toward the tumor (14).

Cytotoxic T-lymphocyte associated protein type 4 (CTLA-4) was described as an inhibitor of T cell activation by Walunas et al. (60). CTLA-4 on APCs prevents CD28/B7 co-stimulation by outcompeting CD28 to interact with B7, thus creating an inhibitory response in the T effector cell (61). However, it also exists on Tregs in a constitutively active form, potentially increasing this cell type's immunosuppressive potency (62). Since the discovery and characterization of CTLA-4, multiple other checkpoints have been discovered including PD-1 (63), LAG-3 (64), TIM-3 (65), CD137 (4-1BB) (66), GITR (67), and CD134 (OX40) (68). These molecular signals have been identified on infiltrating immune cells of many different malignancies, including glioblastoma, and the cancer cells often express the ligands for these checkpoints (69). Programmed cell death-1 (PD-1) is a cell surface immune checkpoint found on effector T cells, while its ligand, PD-L1, can be expressed by glioblastoma cells, with elevated PD-L1 being associated with poor overall survival in glioblastoma patients, independent of other factors (70–72). PD-L1 is additionally found on antigen presenting cells and immunosuppressive immune cells such as Tregs. One study found a correlation between PD-L1 expression and a marker of regulatory T-cells, FoxP3, as well as between FoxP3 expression and patient survival in patients with glioblastoma (73). Lymphocyte-activating gene-3 (LAG-3) is a cell membrane protein found on NK cells, APCs, and some T lymphocytes including Tregs (74). LAG-3 interacts with MHC class II molecules with greater affinity than their typical partner, CD4, and, in doing so, prevents CD4 T helper cell proliferation and cytokine release (60). Groups studying various malignancies have suggested that these checkpoints may work alone or in parallel for immune evasion (75).

## Checkpoint Inhibition

**Figure 2:**
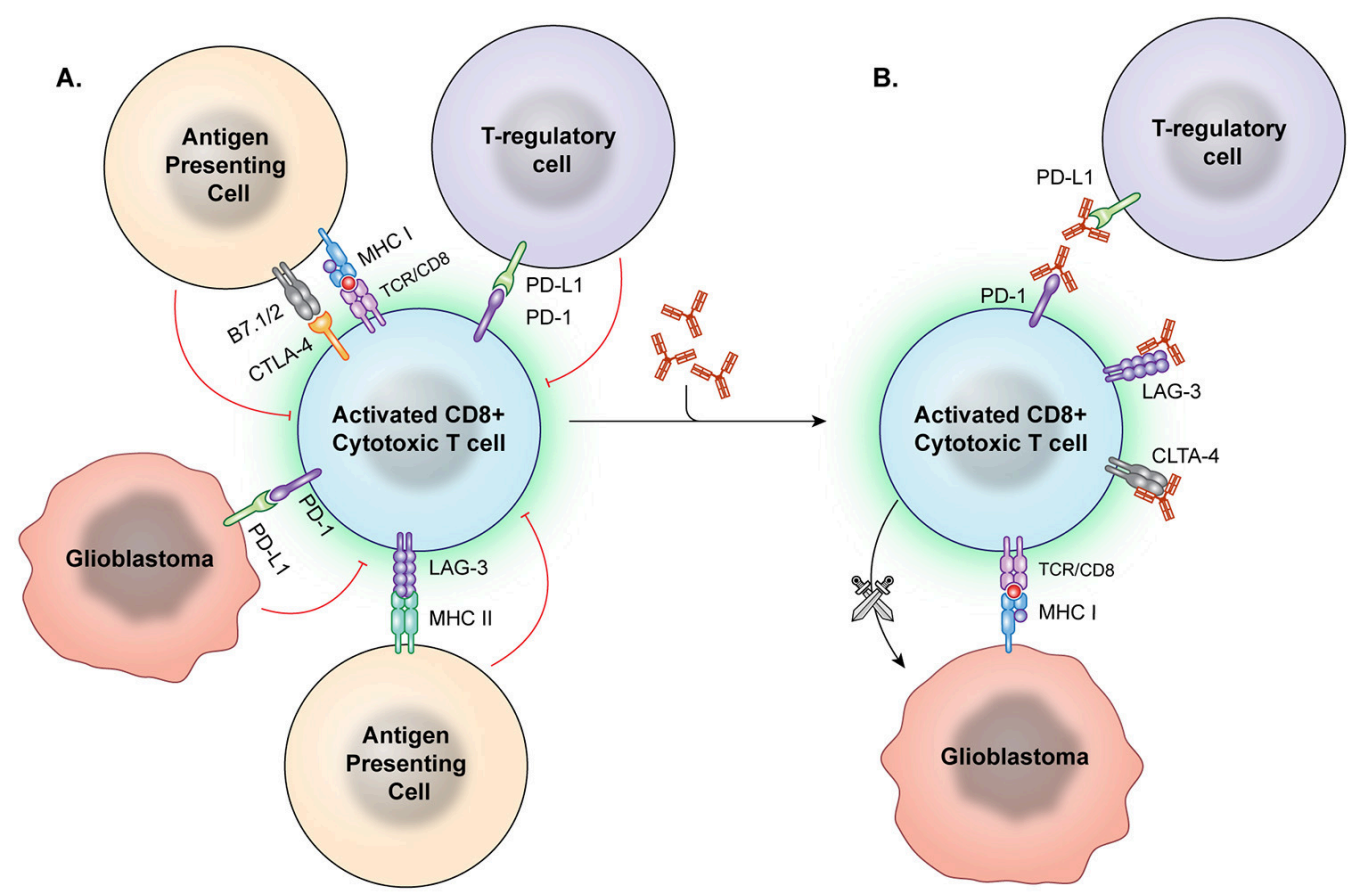
Immune checkpoint inhibition. **(A)** Immune checkpoints hinder T-cell activation and promote an immunosuppressive state. However, these checkpoint molecules can be neutralized by targeted antibodies. **(B)** After the checkpoint molecules are negated by these blocking antibodies, T effector cells are better able to recognize and attack tumor cells.

Checkpoint inhibition describes the use of a treatment to interfere in the interaction between an immune checkpoint molecule and its target, receptor, or ligand (Figure 2). This is intended to cause a net immune stimulating effect by inhibiting an inhibitory signal. After the discovery of CTLA-4's immunosuppressive function, antibody-mediated CTLA-4 blockade caused tumor regression in murine models (54). Following several promising phase II trials (76–78) the first phase III study of a checkpoint inhibitory showed anti-CTLA-4 improved survival in metastatic melanoma in 2010 (79). The Checkmate 067 trial demonstrated that anti-PD-1 and anti-CTLA-4 either alone or in combination imparted increased survival in untreated melanoma. Notably, patients whose tumors displayed increased PD-L1 expression had significantly improved survival on this therapy than those with low PD-L1 expression (10). Similar trends in response to anti-PD-1 therapy related to PD-L1 expression have been described by multiple groups in non-small cell lung cancer (80). However, PD-1 and CTLA-4 pathways may cause immune suppression in parallel, with one trial finding that treatment with either anti-PD-1 or anti-CTLA checkpoint inhibition in melanoma results in upregulation of the other pathway to continue immune evasion, suggesting a potential limitation to monotherapy (81).

Work by groups led by Lesniak and Lim extended these positive findings to glioblastoma in murine models by showing survival benefit in glioma-implanted mice treated with PD-1 blockade in combination with CTLA-4 blockade (82) and with radiation therapy (83). In response to the promising results of checkpoint blockade in non-CNS cancer and what was believed, at the time, to be high expression of PD-L1 in glioblastoma (84) the Checkmate 143, Checkmate 498, and Checkmate 548 trials explored checkpoint inhibition in GBM. The checkmate 498 (85) and 548 (86) trials focused on the use of nivolumab on MGMT unmethylated and methylated newly diagnosed GBM, while Checkmate 143 investigated its use in recurrent GBM (87). Checkmate 143 has completed with no overall survival benefit of nivolumab treatment in this patient population (88). Filley et al. proposed that the negative result may be due to several different factors, including the profound immune suppressive microenvironment of glioblastoma, systemic immune suppression of glioblastoma patients and the antibody's inability to cross the blood-brain barrier (89). Additionally, they postulate that checkpoint inhibition may be less effective at reactivating immune cells rather than preventing immunosuppression in the immune environment in recurrent glioblastoma. Furthermore, steroid use at 4 mg of dexamethasone per day or higher was quite prevalent in the treated population, and the investigators did not obtain pathological confirmation of tumor recurrence prior to halting therapy. This could have led to premature discontinuation of immune therapy, given the difficulty in distinguishing inflammatory treatment response from tumor progression in glioblastoma patients (90). However, there are a variety of other trials investigating the combination of checkpoint inhibition with other therapies, such as vaccination, discussed below, which are still ongoing (85). Potential therapeutic benefit of LAG-3 blockade was shown in a phase I/II trial in metastatic breast cancer (91) and a phase I trial for renal cell carcinoma (92) with a study in pancreatic cancer terminated due to drug production difficulties (93). In response, several phase I studies of LAG-3 blockade are underway, including for a variety of solid tumors (94) and hematologic malignancies (95). One of these studies is currently investigating the use of anti-LAG3 checkpoint inhibition, along with PD-1 and CD137 blockade in recurrent glioblastoma, though results have not yet been released (96).

## Tumor Vaccination

Vaccines have been extensively studied as a potential therapy for gliomas. In general, vaccines expose the immune system to a weakened or killed antigen to build immune memory against any future exposure to that antigen (97). Cancer vaccines work via similar mechanisms. They manipulate immune memory following a primary encounter with a cancer associated antigen to activate T-cells and induce an inflammatory response which is targeted against the tumor.

### Dendritic Cell Vaccines

Dendritic cells (DCs) were first described as novel stellate cells found in lymphoid tissue in 1973 by Steinman and Nussenzweig (98). Our current understanding is that they are intermediate antigen presenting cells (APC) to both CD4+ and CD8+ T-cells as well as activators of natural killer cells (NK-cells) and NK-T-cells in the setting of MHC (96, 99). MHC expression can be downregulated in the glioblastoma microenvironment, reducing the efficiency of antigen presentation (100–102). This makes effective antigen presentation even more critical for successful immune therapy in the brain.

**Figure 3:**
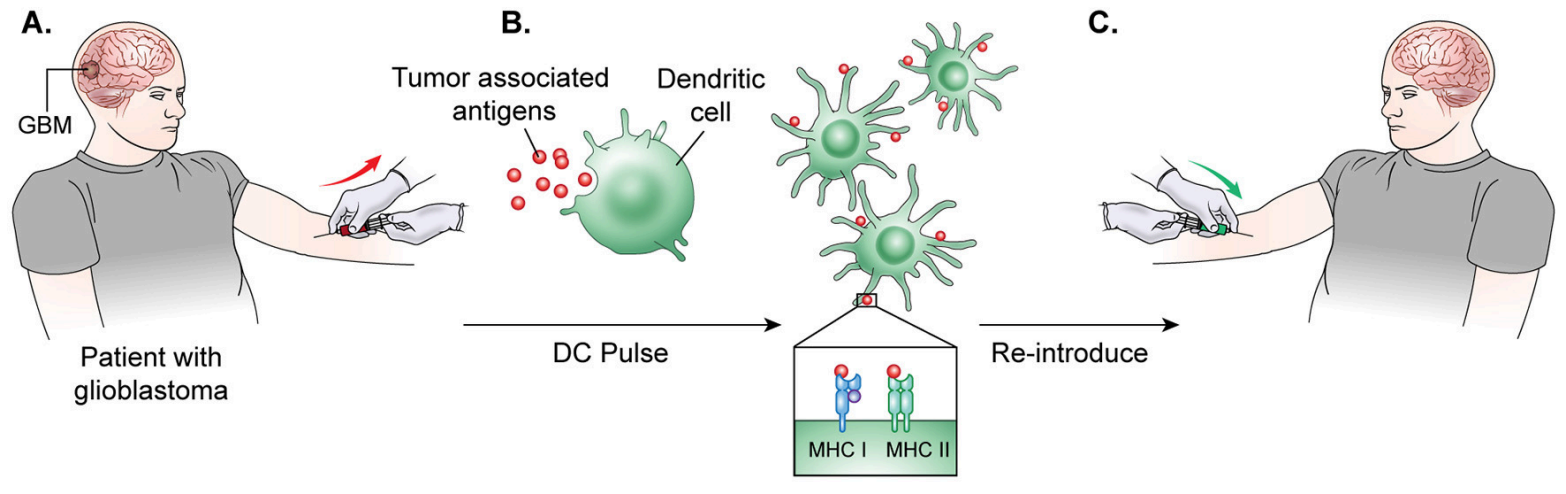
DC vaccine development.**(A)** The general process of DC vaccine development and immunization requires tumor lysate isolation. Patients first undergo resection of the tumor for production of lysate as well as patient leukapheresis to collect dendritic cells. **(B)** The tumor associated antigen, mRNA, or lysate is used to pulse mature or immature DCs obtained through patient leukopharesis. **(C)** Primed DCs are then administered as a vaccine to patients peripherally.

The general process of autologous DC vaccine development requires isolation of DCs from a subject's blood, pulsation of the immune cells with the cancer associated antigen for stimulation, and, finally, treatment of the subject with the newly formed vaccine (103) (Figure 3). Preclinical investigations in mice revealed that peripherally injected DC vaccines could induce cytotoxic T-lymphocyte (CTL) responses in the CNS without causing major adverse effects such as autoimmune responses (104). Further studies in glioma mouse models confirmed that DC vaccines could target and kill these tumors without significantly harming normal brain tissue (105, 106). In a Phase I clinical trial for DC vaccine use in glioma, Wheeler, Wu, and team pulsed immature DCs of 9 glioma patients with tumor peptides eluted from cultured autologous tumor cells (107). Patients underwent initial tumor resection followed by conventional radiotherapy (RT) prior to elution of MHC-I peptides from the tumor sample and DC extraction from host venous blood. They observed that 4 of the 7 patients that were given the vaccine had a positive cytotoxic T-cell response. After vaccination, 4 tumors were re-resected and 2 of them exhibited CD8+ T-cell and memory T-cell infiltration (103). This research illustrated the ability of *ex-vivo* educated DCs to augment the proinflammatory response against tumor.

CD133, also known as prominin-1, is a transmembrane glycoprotein receptor that has been proposed to play a vital role in cell proliferation due its association with the WNT signaling pathway, but its overall function still remains undefined (108). CD133+ cells have been recognized in a study by Singh et al. as potential brain tumor initiating cells. The study showed that only the CD133+ human brain tumor cells possessed the ability to initiate phenotypically identical tumor growth in non-obese diabetic severe combined immunodeficient mouse brains with just 100 cells injected. The CD133- cells on the other hand were not able to cause tumor growth even with as many as 10^5^ cells injected into the mouse brains (109). CD133 is now a common marker used to identify malignant cancer stem cells in GBM (110) as well as in endometrial (111), colon (112), lung (113), prostate (114), ovarian (115), skin (116), and breast (117) cancer. CD133+ cancer stem cells in GBM have been shown to be resistant to radiotherapy and chemotherapeutic drugs (118) as well as contribute to the recurrence of the tumor after radiation (119). A promising DC vaccine trial (120) targeting CD133 was reported in June 2017 at the American Society of Clinical Oncology annual meeting (121). This Phase 1 trial of the ICT-121 DC vaccine was carried out in patients with recurrent glioblastoma who express the HLA-A2 phenotype. In this trial, the patient's DCs were pulsed with CD133 to create the vaccine. The vaccine was then administered to the patient once a week for 4 weeks for the initial induction phase, followed by once every 2 months for the maintenance phase. They reported that the ICT-121 DC vaccine was considered safe and tolerable. Eight out of the 20 patients enrolled were surviving at the time of the report, and cytokine mRNA expression suggested the presence of an active immune response to the CD133 epitopes.

Multiple clinical trials have since been performed to evaluate the safety and efficacy of dendritic cell vaccines in patients. Liau et al. reported Phase I clinical trial results of the use of DCVax-L, a DC vaccine generated with autologous tumor lysate, in newly diagnosed and recurrent glioblastoma patients (122) and they found that the treatment was safe. De Vleeschouwer and team reported in their Phase I/II study feasibility study using a tumor lysate pulsed DC vaccine with RT and concomitant TMZ in 2010, and they demonstrated a 18.3 month median survival with this approach (123). Dr Liau's team has recently reported early results from a Phase III trial of DCVax-L, which completed enrollment in November of 2015. In this study, patients underwent tumor resection for vaccine preparation, they received standard concurrent radiation and chemotherapy with TMZ, and they were subsequently randomized into two groups: a group receiving DCVax-L, and a placebo group (124). Of note, all patients were given the opportunity to receive DCVax-L at the time of progression/recurrence without breaking the blind regarding their initial treatment. As a result, at the time of publication, 86.4% of patients enrolled in the study had received DCVax-L at initial diagnosis or recurrence. The early median survival results show that patients who received the vaccine survived 23.1 months, which does appear promising, but we will be unable to determine the true impact of the treatment until enough events of progression and/or death have occurred to report the unblinded randomized results.

To improve the therapeutic index of these trials, some groups are combining dendritic cell vaccines with other immunomodulatory therapies. Sampson and team in 2014, investigated DC migration to vaccine site-draining lymph nodes following tetanus diptheria toxoid (Td) pre-conditioning in mouse models as well as in patients with newly diagnosed GBM (125). This led to human CMV pp65-LAMP mRNA-pulsed autologous DCs now being used in the Phase II ELEVATE trial (126). Patients with newly diagnosed GBM who have undergone resection and standard TMZ and RT are separated into 3 groups to understand how pre-conditioning the body can affect migration of the pulsed DC vaccine. Groups are given unpulsed DCs, Td, or Td accompanied with the immunosuppressive drug, basiliximab as pre-conditioning for the CMV pp65-LAMP DC vaccine. Basiliximab is a chimeric CD25 monoclonal antibody that has been shown to decrease Treg expansion in transplant patients, and it is being added to the trial to attempt to prevent Treg expansion after TMZ therapy (127).

### Peptide Vaccines

Although DC vaccines have the potential to be effective in patients with GBM, their development poses a challenge to those that may not have the facilities to safely extract and pulse DCs with tumor components, therefore limiting the ability to rapidly scale these therapies for broad utilization. Similar to DC vaccines, peptide vaccines are made from tumor associated antigens, but instead of creating the vaccine in a personalized manner, peptide vaccines are “off-the-shelf” therapies that can be centrally produced. Peptide vaccines are thus more rapidly available for distribution to various medical centers, making them an attractive approach for multicenter trials for glioma immune therapy (128).

A well-studied target for peptide vaccines is the epidermal growth factor receptor (EGFR), a receptor tyrosine kinase that is highly expressed in high grade gliomas compared to normal brain tissue. In GBM, there are frequent mutations in EGFR, with the most common being the EGFR variant III (EGFRvIII) truncated mutant. EGFRvIII does not have a ligand-binding domain like the wild-type receptor, and it is therefore constitutively active, driving tumorigenesis (129, 130). This makes EGFRvIII a very attractive target for immune therapy, as it is a tumor-specific target driver of the malignancy, and it is expressed on the cell surface.

**Figure 4:**
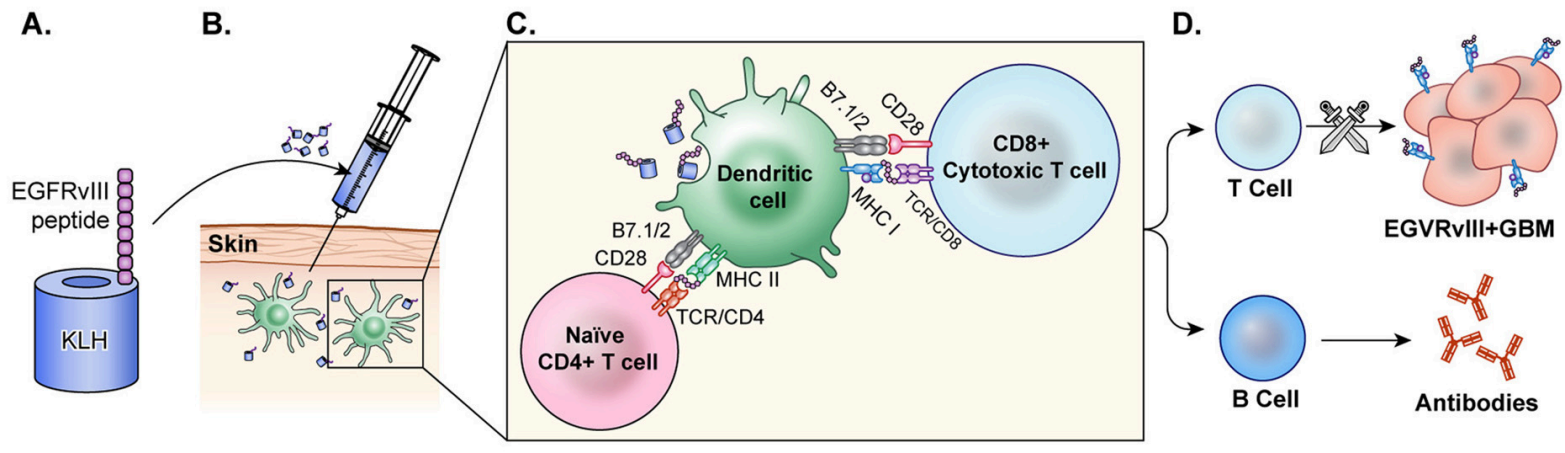
Peptide vaccine. **(A)** In the peptide vaccine, rindopepimut, EGFRvIII peptide is fused with highly immunogenic KLH (PEPvIII-KLH) for vaccine preparation. **(B)** The vaccine is administered intradermally and the antigen is recognized by APCs. **(C)** APCs present to T-cells and CTLs. **(D)** T-cells activate B-cells which then produce antibodies to EGFRvIII in the tumor. CTLs cross the blood brain barrier and target GBM cells with EGFRvIII on the surface. This activation of T-cells and CTLs results in anti-tumor response.

EGFRvIII peptide vaccine was generated by the fusion of a synthetic peptide that represents a truncated amino acid chain of EGFRvIII with keyhole limpet hemocyanin (KLH), a highly immunogenic peptide (Figure 4). Early investigations of EGFRvIII peptide vaccines in murine models resulted in a CTL mediated immune response to the EGFRvIII antigen (131).

John Sampson, Amy Heimberger, and team conducted initial trials using rindopepimut, a peptide vaccine targeting EGFRvIII, in glioblastoma patients. They observed that the drug is tolerated at maximal doses with minimal adverse effects as determined by the Phase I VICTORI trial (132). In the Phase II ACTIVATE trial, they administered rindopepimut to newly diagnosed GBM patients who had undergone surgical resection and were receiving TMZ treatment. They showed a significant increase in both PFS and mOS at 14.2 and 26 months, respectively, with minor adverse reactions (133) which suggested that rindopepimut was both safe and beneficial to use with the standard GBM treatment of TMZ.

TMZ, however, can cause myelosuppression and lymphopenia in patients undergoing the treatment (134). ACT-II, a Phase II clinical trial of rindopepimut, revealed that EGFRvIII peptide vaccine could still be beneficial in conjunction with the standard treatment of TMZ in newly diagnosed GBM patients. However, they showed that at higher doses of TMZ, patients were at risk for greater toxic effects (135). Additionally, targeted tumor cells lost expression of EGFRvIII, demonstrating the specificity of the drug for its target (120).

The positive results observed in both the ACTIVATE and ACT-II clinical trials allowed for an expansion to a multicenter Phase II investigation of the drug known as ACT-III. Sixty-five newly diagnosed GBM patients were treated with rindopepimut and concomitant TMZ and the results continued to show increased PFS and mOS, as well as increased anti-EGFRvIII antibody titers in 85% of the patients (136). The vaccine then became part of a global, double-blind Phase III clinical trial in newly diagnosed GBM cases [ACT-IV 137).Unfortunately, ACT IV was terminated in 2016 as the control group had a higher mOS than the vaccinated group. It is believed that, in part, the heterogenous expression of EGFRvIII in GBM may have played a role in the failure of the trial (138).

The ReACT Phase II clinical trial used a combination of rindopepimut with the VEGF monoclonal antibody, bevacizumab, in patients with recurrent GBM, as bevacizumab has been shown to improve PFS in recurrent GBM (139). This trial showed a positive trend toward increased PFS in recurrent GBM patients (140).

Aside from targeting EGFRvIII, ongoing clinical trials are utilizing vaccines targeting multiple antigens, such as the 11 tumor-associated peptides targeted by the IMA950 trial. In this study, the peptide vaccine is given together TMZ and either Poly-ICLC (141) a vaccine with broad innate and adaptive immune enhancing effects, or RT (142). This multi-peptide approach could overcome the antigen loss often seen when targeting a single tumor-associated antigen (120).

### Heat Shock Protein Vaccination

**Figure 5:**
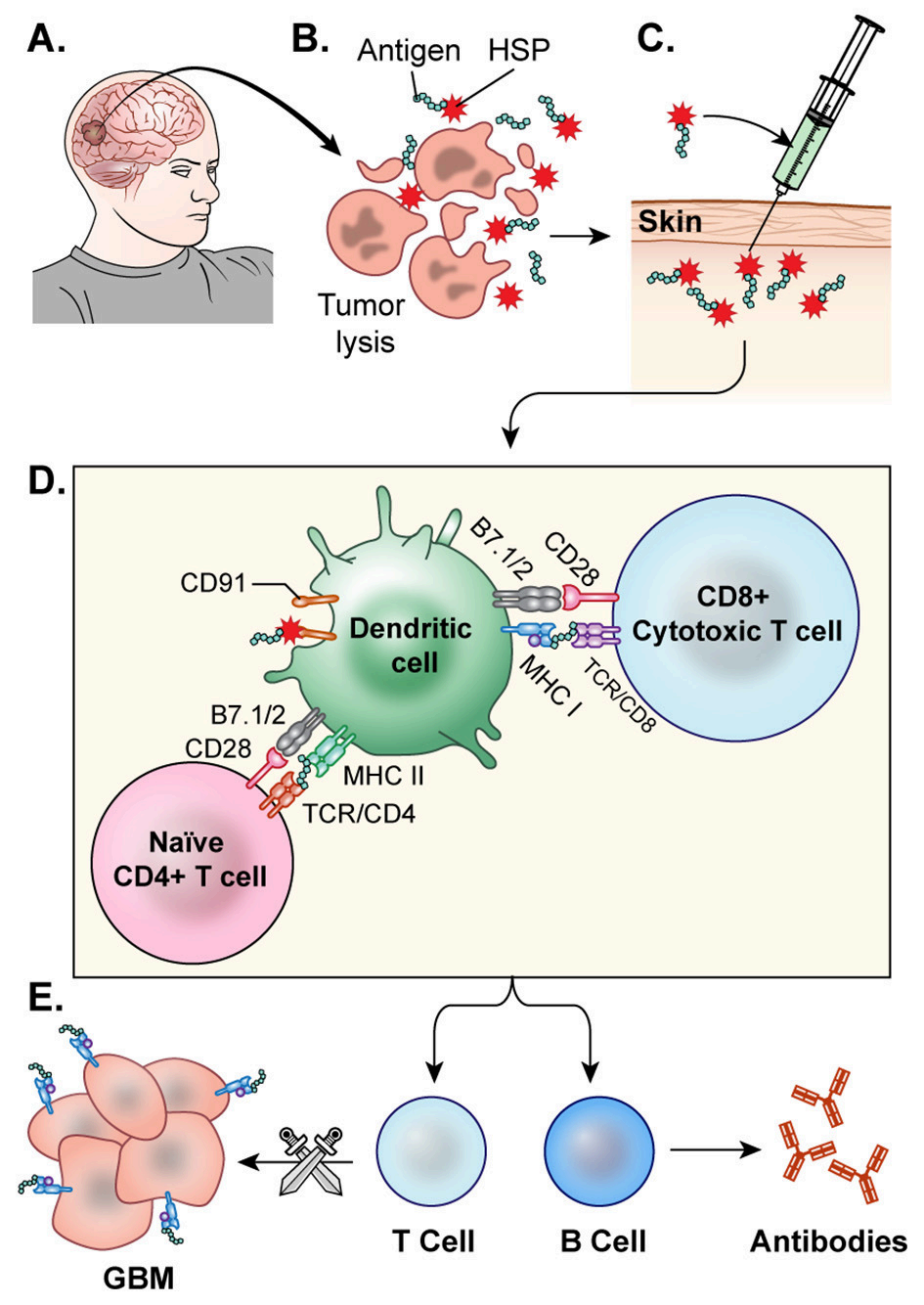
HSP vaccine. **(A)** The glioblastoma is resected. **(B)** HSPs bound to the tumor antigen are released by *ex-vivo* tumor cell lysis. **(C)** The desired HSPs are isolated and peripherally administered back to the patient as a vaccine. **(D)** Once injected, the HSP-tumor peptide complexes are taken up by antigen presenting cells, likely facilitated by CD91, and these peptide complexes are presented on MHC Class 1 molecules for recognition by CTLs **(E)** CTLs cross the blood brain barrier and target GBM cells. This activation of T-cells and CTLs results in anti-tumor response.

Heat shock proteins (HSPs) can broadly activate both the innate and adaptive immune systems as well as enhance MHC-I and MHC-II presentation of antigens (143). Tumor cells have increased HSP expression because of their high metabolic rate, which can leave the cells riddled with misfolded and aberrant proteins, resulting in cellular stress (144). HSPs make complexes with cellular stressors such as antigens and traffic them to APCs, where they can ultimately induce an anti-tumor immune response, making them an attractive component in cancer vaccine development. The vaccine is generated following resection of a patient's tumor. HSPs released from the resected tumor cells are believed to form complexes with tumor associated antigens. The HSP-tumor peptide complexes are isolated from the tumor, *ex vivo*, verified via Western Blot, and purified to make the vaccine. Then, they are peripherally administered back to the patient, where it is hoped that these HSP complexes will help prime CTL against the tumor (145) (Figure 5). Initial investigations utilizing HSP peptide complex-96 (HSPPC-96), an HSP that can bind tumor-associated antigens, as a tumor vaccine revealed that this treatment was safe and there was significant peripheral immune response to the treatment (146). This phase I trial, led by Andrew Parsa and Orin Bloch, disclosed a significant immune response specific to the tumor site in 11 of the 12 recurrent GBM patients they treated with the vaccine with minimal adverse effects. The mOS for those 11 responders was 47 weeks post-surgery and vaccination compared to 16 weeks of the single non-responder (128). In a follow-up Phase II trial, the team revealed the safety and efficacy of the vaccine when used with concomitant standard TMZ therapy (147). The group further demonstrated that PD-L1 expression on migrating myeloid cells induced systemic immunosuppression that could diminish the effect of the vaccine. Their 2009 Phase II HeatShock trial in newly diagnosed GBM demonstrated that MGMT methylation, Karnofsky performance score (KPS), and PD-L1 expression were prognostic factors for vaccine effectiveness (148). Mark Gilbert and team are currently recruiting newly diagnosed glioblastoma patients for a Phase II clinical trial investigating the effect of HSPPC-96 vaccine treatment together with standard radiotherapy and TMZ as well as pembrolizumab, an anti-PD-1 checkpoint inhibitor, to elucidate whether the HSPs from these individuals can enhance pembrolizumab efficacy (149). Dr. Fangusaro is also investigating this approach in pediatric subjects diagnosed with either HGG or ependymoma. They aim to determine if the vaccine is both efficacious and safe in the pediatric population (150).

## Adoptive Cell Therapy (ACT)

Adoptive cell therapy (ACT) has recently emerged as a promising immunotherapeutic regimen against various malignancies. ACT refers to the collection and manipulation of a patient's lymphocytes to target and kill cancer cells, first described when Delorme et al. observed that transferred lymphocytes could inhibit proliferating sarcomas in rat models (151).The first report of this approach for human use was in 1988 by Rosenburg et al. They used tumor-infiltrating lymphocytes (TILs) in combination with interleukin-2 to treat metastatic melanoma. In this study, they found objective regression in 60% of patient tumors. TIL treatment is now considered a highly effective therapy against this disease (152). Since then, ACT has advanced and now embodies a broad scope including several treatment modalities: tumor-infiltrating lymphocyte (TIL) immunotherapy, T-cell receptor (TCR) therapy, and chimeric antigen receptor (CAR) T-cell therapy.

### TIL Therapy

The use of TILs, infiltrative lymphocytes with increased tumor-specificity that reside within the peritumoral space, for cancer therapy was introduced by the Surgery Branch at the NIH. This treatment involves TIL harvest at the time of tumor resection, expansion and stimulation with IL-2, and reinfusion of stimulated TILs into the body (153). Rosenburg et al. demonstrated the success of this treatment in metastatic melanoma (154). TIL therapy resulted in a complete response in approximately 20% of metastatic melanoma patients, with 40–50% of patients exhibiting transient response (155, 156). Solid tumor regression using TILs has also been reported in ovarian cancer and renal cell carcinoma (157). Additionally, TILs have recently gained popularity because these cells can be modified to recognize tumor-specific mutations creating neo-epitope reactive TILs (158). This treatment was applied in patients with colorectal cancers using TILs specifically targeting the Kras G12D mutant (159). Next generation sequencing to identify tumor-specific mutations shows promise in generating a genetically precise model of tumor-targeted TILs (160, 161) TIL therapy may be a promising mode of treatment for glioblastoma, but TIL therapy for the treatment of CNS malignancies has to be carefully investigated for its potential toxic effects and risk of cytokine release syndrome (162). Overall, recent technological advances in ACT expansion methods and preconditioning show progress for this field, but difficulties pertinent to treating central nervous system malignancies remain.

### TCR Therapy

TCR therapy uses patient T-cells obtained from the peripheral blood which are modified to express tumor-specific α and β chains for enhanced antigen-recognition in treating solid tumors (163). TCRs express naturally occurring receptors that can recognize surface antigens as well as intracellular tumor antigens on antigen presenting cells by binding to the MCH complex (164, 165). The most notable clinical applications include the use of TCR therapy to target melanoma-associated antigen recognized by T-cells 1 (MART-1), melanoma-associated antigen A3 (MAGE-A3), and New York esophageal squamous cell carcinoma antigen (NY-ESO-1) (166–168). These trials demonstrated at least partial responses in patients with metastatic melanoma and synovial sarcoma and continue to show promise (148–150, 169). An ongoing trial is recruiting patients with diverse cancers including melanoma, synovial sarcoma, breast cancer, and non-small cell lung cancer patients for TCR directed NY-ESO-1 therapy (170, 171). While TCRs targeted to these specific antigens have not been tested in GBM, these antigens do present in gliomas as well, and due to their efficacy in other cancers warrant further investigation (172, 173).

### CAR T-Cell Therapy

**Figure 6:**
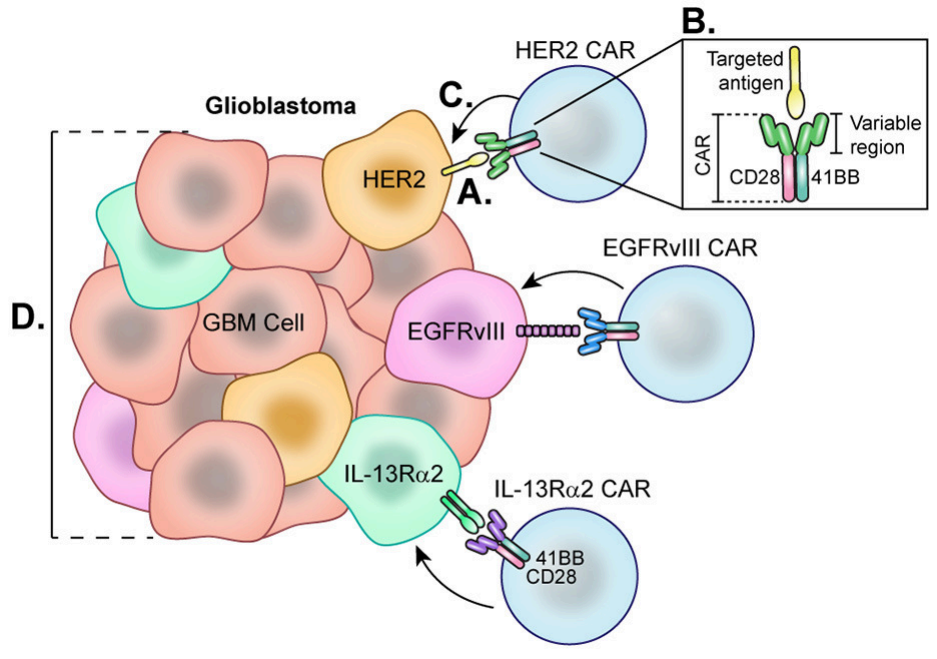
Chimeric Antigen Receptor (CAR) T-cells each directed at a specific GBM-specific tumor antigen. Each CAR T-cell therapy developed for the treatment of glioblastoma utilizes a CAR directed toward one antigen such as HER2, EGFRvIII, or IL-13Rα2. **(A)** Engagement of tumor-specific CAR T-cells with target cell surface antigen present on tumor cells causes CAR T-cell activation. **(B)** Second and third generation CAR T-cells are synthesized with co-stimulatory molecules such as 41BB, CD28, and CD3 which lower the CAR T-cell barrier to activation. **(C)** Fully activated CARs attack target cells causing tumor cell lysis. **(D)** Cells negative for the CAR T-cell target demonstrate the heterogeneity of the tumor and represent a barrier to treat as these cells not targeted continue to proliferate.

In CAR T-cell therapy, T-cells obtained from peripheral blood of patients are genetically engineered to express synthetic chimeric antigen receptors (CARs) on their cell surface which are specific for antigens expressed on a tumor's cell surface (174). These cells are then expanded *in vitro* and returned to the patient via infusion to subsequently proliferate in the body (175). Further modification and enhancement of CARs has generated a new generation of these synthetic T-cells. First-generation CARs are designed simply with an antigen recognition domain. However, second-generation CARs and beyond have additional co-stimulatory domains such as CD28 and 4-1BB which lower the barrier to activation and optimize receptor function (176) (Figure 6). CAR T-cell therapy has had success in treating various blood cancers by targeting an antigen, CD19, which is expressed among B-cells (177). Specifically, successful clinical trials led by Grupp et al. treating acute lymphoblastic leukemia (ALL) using CD19-targeted CAR T-cells led to its FDA approval in 2017 (178). However, disease recurrence has been observed in various clinical trials using CD19 CAR T-cell therapy to treat leukemias and lymphomas due to a phenomenon of antigen loss, where cancer cells no longer express CD19 (179). CD19-targeted CAR T-cell therapy has since been modified to address antigen loss by altering CARs to express antigen receptors that bind CD22 or CD123, which are antigens also expressed by these neoplastic B-cells (180). Additionally, Kochenderfer et al. developed a CAR T cell therapy targeted to B-cell maturation protein (BCMA), present in a majority of multiple myeloma (MM) cells, which resulted in a complete response in 50% of patients for this ongoing trial (181).

With the overt success of ACT treatment against blood cancers, questions have arisen about the potential efficacy of CAR-T-cells against solid tumors. Some in the field are skeptical that CAR T-cell therapy will demonstrate the same efficacy in solid tumors because the majority of solid tumor antigens exist inside the cell, away from CAR T-cell binding (182). However, investigators have made progress in generating CAR T-cells that target proteins overexpressed in many solid cancers with promising results in preclinical models of pancreatic cancer (183) neuroblastoma (184) and lymphoma (185) among others. As a result, phase I and phase I/II trials are underway targeting a variety of antigens in solid tumors (186).

For GBM, CAR T-cells generated to target one of three GBM-specific antigens—HER2, IL-13Rα2, or EGFRvIII—are being studied in clinical trials. HER2 is a tyrosine kinase receptor overexpressed in many cancers, including up to 80% of GBMs (187) (Figure 6). In 2010, a phase 1 trial was started at Baylor using CAR T-cells targeting HER2. Of note, these CARs were generated with a CD28 signaling domain and pre-selected for their ability to naturally recognize cytomegalovirus (CMV), which may augment therapeutic potency of these cells by also targeting CMV-related peptides in the glioblastoma microenvironment. A total of 16 recurrent HER2^+^ GBM patients were enrolled for treatment by this second generation HER2-CAR CMV-T cell which led to a partial response in 1 patient lasting more than 9 months. Of the 16 patients, 7 presented with stable disease for up to 29 months. Blood samples revealed that HER2-CARs were detected in only 7 patients at 6 weeks post-treatment, and levels continued to decline, suggesting these CARs were unable to expand *in vivo* (188). Although this therapy showed early signs of efficacy, one challenge is prolonging the life of these CAR T-cells. Beginning in 2018, City of Hope is leading a phase 1 clinical trial by Dr Badie and colleagues that aims to treat HGG patients with autologous memory-enriched T-cells transduced via lentivirus to express HER2 and 41-BB co-stimulant (189) as 41-BB has been reported to improve CAR T-cell persistence (190). The addition of this costimulatory signal has led to improved cytotoxicity to the target tumor cells (191, 192) as well as decreased T cell exhaustion (193) in *in vitro* models.

IL-13Rα2 is another promising antigen expressed in approximately 75% of GBMs but not at significant levels in normal brain cells (194) (Figure 6). From 2008 to 2011, Badie and others at City of Hope held a pilot safety and feasibility trial which enrolled 3 HGG patients for treatment using first generation CAR T-cells directed at IL13Rα2 via intracranial delivery directly to the resection cavity. Patients were not excluded based on lack of IL13Rα2 antigen positivity. Overall, all 3 HGG patients showed decreased IL13Rα2 tumor expression following therapy, and the post-relapse mean survival was 11 months (195). City of Hope then began using second generation IL-13Rα2-41BB co-stimulated CAR T-cells to treat recurrent or refractory HGG patients using intracavitary, intratumoral, or intraventricular infusions (196). While this study is still recruiting and final results have not been reported, a case study from this trial demonstrated complete response of recurrent multifocal GBM lasting for 7.5 months (197). This patient was treated with intracavitary infusion until leptomeningeal disease progression was found, at which point CAR T-cells were administered by intraventricular infusion which led to transient complete response, though notably, recurrence ultimately occurred in the form of distal metastases that expressed lower levels of IL13Rα2. The promising results of this work, though limited by small patient numbers, encourage further investigation.

Lastly, the EGFR variant, EGFRvIII, is a tumor-specific truncated version of the EGFR receptor, making it an attractive glioblastoma cell-surface target for CAR T cells. There are currently six ongoing trials that utilize CAR T-cells directed at EGFRvIII. Beginning in 2011, Rosenberg et al. of the National Cancer Institute led the first clinical trial directed at EGFRvIII, which is still ongoing (198). Early success has been reported in a phase I clinical trial of EGFRvIII-targeted CARs led by Donald O'Rourke and colleagues at the University of Pennsylvania. Of the 10 patients, 7 patients underwent reresection after therapy. Tissue from 3 of these patients demonstrated reduction of EGFRvIII expression, and 2 had complete elimination of detectable expression. Additionally, they noted that 3 patients demonstrated lymphocytic tumor infiltrates with broad T cell clonotype diversity. However, progression occurred in almost all cases despite antigen loss. As of 2017, one patient survived greater than 18 months with no further treatment while another two patients are alive, albeit with signs of disease progression. Importantly, the tumor microenvironment of surgical specimens from CAR T-Cell treated tumors in this trial displayed marked upregulation of PD-L1, IDO, and TGF-β, as well as FoxP3+ Tregs (199). This suggests that tumors adapt to treatment with this CAR T-cell and are capable of immune escape by activating various immunosuppressive pathways. Further investigations with *in vivo* animal models suggest that a durable anti-tumor response to CAR T cell therapy can be elicited by targeting this reciprocal immunosuppression with other immune-modulatory therapies (200–202) (Figure 6).

There are many complexities to treating GBM with CAR T-cells. Barriers to durable responses include the lack of long-term CAR T-cell persistence; ineffective delivery of cells to the infiltrating tumor; and GBM's characteristic immunosuppressive properties. Several of these barriers are being addressed in ongoing trials. For example, Memorial Sloan Kettering has created “Armored” CAR T-cells which constitutively secrete IL-12, a potent pro-inflammatory cytokine for cytotoxic T cells. *In vitro* work has shown that this IL-12 expression induces enhanced proliferation, persistent cytotoxicity, and decreased apoptosis of CAR T-cells (203).

## Viral Therapeutics

**Figure 7:**
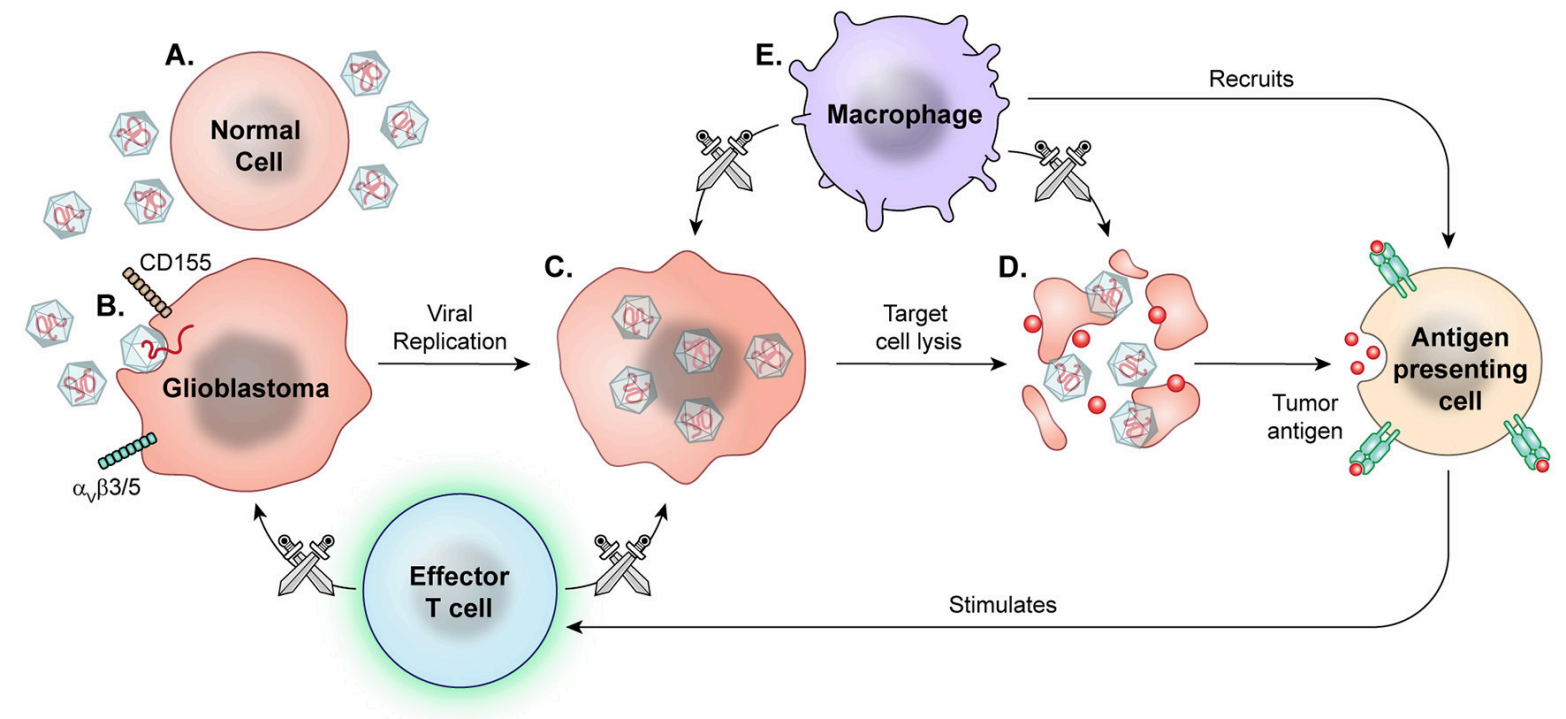
Prototypical mechanism of oncolytic viral therapy. The modified virus is infused into the tumor environment. **(A)** Normal cells exposed to viruses may have introduction of viral genetic information, but the viruses are modified to not replicate. **(B)** Viral particles then recognize enter cell based on specific surface proteins, such as CD155 in PVSRIPO and α_V_β_3/5_ in Delta-24-RGD oncolytic adenovirus. **(C)** Oncolytic viral particles in tumors are replication-competent and recruit tumor cell replication machinery. **(D)** Viral replication results in cell lysis and release of viral particles to continue targeting tumor cells. **(E)** Macrophages detect and target virally infected cells, recruiting other APCs and effector T cells for secondary immune response against released tumor antigens.

Viral therapy has undergone extensive research over two decades, with some promising results in both pre-clinical and early clinical trials. Viral therapies use replication competent, albeit attenuated or genetically modified, viral species, taking advantage of both oncolytic and non-oncolytic mechanisms for high grade glioma targeting (Figure 7).

Delivery of these viral therapeutic agents provides a practical challenge given the blood-brain barrier and the need to provide sufficient concentrations of agent at the site of action while minimizing risk of systemic toxicity. Convection enhanced delivery (CED) has been utilized to overcome these delivery challenges. CED, first described by Oldfield et al. in 1994, utilizes catheters to directly infuse a therapeutic agent to the central nervous system (204). This technique has been applied to glioblastoma treatment in a limited group of clinical trials, including the PRECISE trial. The PRECISE trial is, to date, the only completed phase III evaluation of convection enhanced delivery for the treatment of glioblastoma. The trial utilized cintredekin besudotox, a chimeric cytotoxin composed of a mutated *Pseudomonas aeruginosa* toxin attached to recombinant interleukin-13. Delivered by CED, this cytotoxin targets cells expressing the IL-13 receptor. They reported increased progression-free survival but not an increased overall survival compared to implanted Gliadel wafers in recurrent glioblastoma (205). This trial's failure may be attributed to less than 70% of catheters being placed according to protocol. Also, the statistical power study was designed such that although the CED group met the overall survival cutoff needed for significance, the control group had a significantly longer survival than expected, decreasing the difference in results of the two arms. These limitations highlight the difficulty of designing trials for CED in glioblastoma patients. However, other *in vivo* preclinical and early clinical studies have demonstrated feasibility of CED with other chemotherapeutic agents (206–209).

CED has been established as a viable technique for infusing viral therapeutics into the brain. Numerous groups have investigated a variety of genetically modified viruses *in vitro* and in non-human *in vivo* models via direct oncolytic activity or as a vector. Candidates include herpes-simplex virus (210) measles (211, 212) JC virus (213) Zika virus (214) adenovirus (192, 215–217) and pox virus (218) , among others. The combination of viral therapy with traditional chemoradiation is also under investigation (219, 220). Following the encouraging results of Delta-24-RGD oncolytic adenovirus in an immunocompetent murine glioma model (221) a phase I trial of the virus infused via CED into recurrent malignant gliomas was completed. The authors reported a subset of patients achieving long-term survival. This study was designed with two groups, one undergoing viral therapy alone, the other undergoing therapy followed by resection 2 weeks later in order to study the tumor tissue after undergoing treatment. They found that in the tumors resected 2 weeks after treatment, there was still active viral replication, while this was not seen in tumors resected at 6 weeks and 2.5 years post treatment. Additionally, at time of tumor resection, after receiving adenoviral therapy, the group resected 4 weeks after therapy displayed enhanced immune cell infiltration and decreased TIM-3 expression. As TIM-3 expression on T-cells is considered a marker for T cell exhaustion, this finding suggests improved T cell functioning in the treated tumor microenvironment. Based on the increased immune response at 4 weeks relative to 2 week samples in this trial, the investigators concluded that tumor regression reflects a delayed secondary immune response rather than direct oncolytic activity (222).

The recombinant polio-rhinovirus chimera (PVSRIPO), developed at Duke University, takes advantage of the poliovirus receptor CD155 found on many neoplastic cells. Preclinical studies demonstrated tumor regression in murine models of breast and prostate cancer treated by PVSRIPO (223). Similarly, treatment of preclinical murine glioma models with this viral therapy has led to tumor regression (224, 225). A phase I trial of PVSRIPO infused via CED in biopsy-confirmed recurrent glioblastoma showed a modest improvement in overall survival, but significant improvement in long term survival, with 21% of patients surviving at 24 and 36 months (226). While this study is limited by the use of historic controls, it warrants further study.

## Future Directions and Biomarker Development

Even in the most successful trials of immune therapy for cancer, these treatments tend to be effective only in a subset of the treated patients. This highlights a need for better biomarkers that might predict which patients will be responders to immune therapy or could direct clinicians to other treatments that might augment the responses of predicted non-responders. In other cancers, there has been some limited progress in the identification of biomarkers of response to immune therapy. Wargo et al. illustrated the complexity of monitoring immune therapy response in melanoma patients, noting changing biomarker expression in longitudinal tissue samples over the course of treatment with serial checkpoint inhibitor monotherapy (77). Interferon-γ pathway loss has been associated with resistance to CTLA-4 blockade in melanoma (227) making it a potential biomarker of immune evasion and poor response to therapy. PD-L1, however, has had mixed reports as a tissue marker of potential therapeutic response. High expression has been associated with improved response to checkpoint inhibition in metastatic Merkel cell carcinoma (228) and squamous cell carcinoma (229). However, it does not appear to be predictive in non-small cell lung cancer (230) and metastatic urethral cancer (231). Additionally, Wolchok et al. reported from a phase III study of PD-1 and CTLA-4 blockade in melanoma that PD-L1 appeared to be prognostic of improved overall survival rather than predictive of checkpoint blockade therapeutic response (232). In all, current understanding of biomarkers is inconsistent and incomplete.

In glioblastoma patients, immunophenotyping has, thus far, been limited in that it typically relies on repeat tissue sampling. While stereotactic biopsies have relatively low morbidity, repeat biopsies are not considered standard practice in glioma clinical trials. This lack of sampling raises additional challenges, as it is quite difficult to determine if a glioblastoma patient has had response to immune therapy on imaging alone. Inflammatory responses to immune therapy often cause contrast enhancing lesions in the brain that can be difficult to distinguish from tumor progression on MRI (86). These limitations necessitate the identification of biomarkers to determine candidates for immune therapy and to track response reliably. The Checkmate 143 trial measured PD-L1 expression of patients entering the trial in an attempt to identify responders, but the full results of this trial have not yet been reported. Other trials have reported biomarkers in tissue pathology following tumor resection after treatment. Lang et al. reported increased TIM-3 expression on T-cells after treatment with Delta-24-RGD, suggesting improved T cell functioning (226). O'Rourke et al. reported upregulation of PD-L1, IDO, and TGF-β, as well as FoxP3+ Tregs in resected tumors following EGFRvIII CAR T-cells, potentially reflecting tumor adaptation and immune evasion (181). However, less invasive serum biomarkers have also been used effectively. In the phase 3 ACT-IV Trial for rindopepimut, investigators used serum titers for antibodies to EGFRvIII to monitor degree of host immune response to the vaccine after administration (119). In other studies, PD-L1 expression on circulating macrophages, and pathological response to therapy have been used to determine whether a response has occurred. In the phase II trial of autologous head shock protein peptide vaccination for glioblastoma, Bloch et al found that patients with low PD-L1 expression on peripheral myeloid cells had on overall survival of almost 45 months compared to 18 months in those with high expression (130). Non-invasive serum biomarkers such as soluble PD-L1 (233) cytokines (234) and peripheral mononuclear cells (235) have been described in monitoring response to immune therapy in non-CNS tumors (236) but similar studies have not been performed extensively in glioblastoma. However, additional biomarkers need to be developed to have a more complete understanding of host and tumor response.

The use of microdialysis in neuro-oncology is a promising addition to our arsenal for immune monitoring of glioma patients. Early use of cerebral microdialysis predominantly focused on patients with neurologic trauma (237–242) or subarachnoid hemorrhage (243–248) demonstrating safety and viability of the technology. More recently, this technique has been applied to brain tumor patients. Portnow et al. utilized microdialysis to monitor in real time if treatment with chemotherapy changed levels of 17 cytokines compared to craniotomy alone. This study showed that craniotomy induced an inflammatory response that dissipated over the next 96 h after surgery (249). Tabatabaei et al. utilized microdialysis catheters in peritumor tissue in high grade glioma patients undergoing radiation therapy, finding that treatment induces a strong inflammatory response via macrophages and monocytes (250). The development of checkpoint inhibitors, though demonstrably effective in metastatic melanoma and non-small cell lung carcinoma, has had mixed results human glioma studies thus far. Understanding the immune profile of the glioma microenvironment in patients undergoing this therapy may allow for patient stratification to determine those with the greatest potential for therapeutic benefit. The ongoing study “Cytokine Microdialysis for Immune Monitoring in Recurrent Glioblastoma Patients Undergoing Checkpoint Blockade” uses microdialysis for monitoring of immune functioning in the glioblastoma tumor environment following checkpoint inhibition. Concurrently, the study samples serum, CSF, and bone marrow for comprehensive analysis of potential biomarkers indicating response to therapy (251).

### Sequencing and Biomarker Development

Advances in understanding the genetics of glioblastomas has led to discovery of prognostic and predictive factors as well as potential targets. Initially, the identification of 4 different molecular profiles (252) of GBM expanded understanding of pathology of the disease beyond histology. Subsequent identification of isocitrate dehydrogenase (IDH) mutation and MGMT methylation statuses and their association with survival (253, 254) as well as response to chemotherapy and radiation (255–257) have proven invaluable to patient care and stratification for appropriate therapy. As technology and methodology have improved, DNA and RNA sequencing allow further mutation identification (258) including biomarkers with associated survival implications (259, 260). Additionally, these techniques may now be able to move out of the laboratory into the clinical setting as sequencing technology becomes more accessible for use in patient care. Kazimierz has shown that whole-genome and RNA sequencing can be performed in a timely and efficient manner (261). As sequencing has become more efficient, its use in guiding clinical decision-making has become feasible, with Byron et al finding that results could consistently be obtained within 35 days of surgical resection (262). Pertinent to this review, sequencing may have implications for immune-based therapies. Early sequencing of common *in-vitro* glioblastoma cell lines identified which lines expressed HLA subtypes associated with improved antigen presentation and response to immunotherapy (263). Song et al. then expanded on similar sequencing work in 298 glioblastoma and control patients, identifying HLA subtypes associated with decreased tumor incidence (264).

Evidence from non-CNS malignancies has suggested an association between the number of somatic mutations identified via sequencing of malignant cells (mutational burden) and the cancer's relationship with the host immune system. Mutational burden is widely variable between malignancies (265) with subsequent groups demonstrating these mutations act as neoantigens to target in adoptive T-cell therapy (266) as well as checkpoint blockade (267). Cancers that have had promising results with checkpoint blockade in human trials, thus far, have been associated with high mutational burden, such as melanoma (268) and non-small cell lung cancer (269). However, further investigation suggests that degree of mutation burden has not had a strong association with response to checkpoint blockade across a broad cross section of malignancies (270). A pilot study reported two patients with glioblastomas due to bi-allelic mismatch repair deficiency, which results in high mutational burden. They found promising results in response to checkpoint inhibition, describing both clinical and radiographic improvement (271). However, despite the inter and intra-heterogeneity of the genomics of glioblastoma, Hodges et al found that only a minority of these tumors were found to have a high mutational burden, and the degree of burden did not correlate with immune cell infiltration into the tumor (272).

As mutational burden alone has not been a strong predictor, other groups have investigated specific somatic mutations, such as the interferon-gamma pathway that may portend a decreased response to checkpoint inhibition (273) in non-CNS tumors. Similarly, in glioblastoma, a diverse population of tumor-infiltrating lymphocytes, thought to more effectively combat a heterogeneous tumor, may not be dependent on intratumoral genomic heterogeneity (274) but rather in 23 genes in immune-related pathways whose expression was found to be significantly associated with prognosis via The Cancer Genome Atlas GBM dataset (275). Goodman et al analyzed a de-identified tumor database, finding that solid tumors with PD-L1 amplification in their genomic profile had almost 70% reported response to checkpoint inhibition, including in glioblastoma. This study again demonstrated that in these tumors, overall tumor mutational burden was only low-to-intermediate (276). As immune-relevant mutations have been identified, comprehensive testing of these mutations is being implemented in clinical decision-making. Peng et al utilized exome, whole genome, and RNA sequencing to identify glioblastoma patients that are least likely to respond to traditional therapy and may be candidates for immune therapy (277). Similarly, Chen et al. categorized multiple malignancies, including GBM, based on tumor microenvironment immune types, to identify genetic patterns that have the greatest potential for therapeutic response to immune therapy (278). While not yet described in GBM, sequencing of circulating tumor DNA in the serum has identified single nucleotide polymorphisms in PD-L1 genes that correlate with improved response to PD-1 blockade in NSCLC (279).

### Sequencing and Development of Patient-Specific Therapeutics

Beyond identifying patients who may have the greatest benefit from immune therapy, Tran et al. reported using neoantigens identified via sequencing as targets for adopted T cell transfer with promising results in non-CNS cancers (141, 280). Monovalent and polyvalent vaccination, described previously, may incorporate these additional targets as more are identified (281). Dunn et al used whole exome and RNA sequencing to identify potential tumor neoantigens in murine glioma models, then use ELISPOT to screen for immunogenicity. These techniques allowed the identification of targets that infiltrating tumor CD8 cytotoxic T cells recognized and bound with high affinity. They suggest that this view into the function of the endogenous immune response to a tumor may provide guidance in the development of personalized tumor vaccination (282). As these lines of research continue, there is great potential for both the development of novel therapeutics as well as the identification of the patients who will most benefit.

### Bispecific T-Cell Engagers (BITEs)

First described in 1961, bispecific antibodies have two variable segments allowing for binding two antigens (283). Nitta et al reported the use of bispecific antibodies to coat activated lymphocytes with partial response in a subset of 10 high-grade glioma patients (284). Bispecific antibodies would be the precursor to bispecific T-cell engagers (BITEs). In 1985, Staerz reported the use of bispecific antibodies in which one of the variable segments targeted the T-cell receptor with the goal of recruiting T-cell mediated immune response (285).

In 1995, Mack et al used BITEs to induce effective cytotoxicity in a cell line transfected to express the target antigen (286). The first drug of this type, blinatumomab, used segments specific for CD19 and CD3 to facilitate T cell targeting of leukemia. It was successful in several phase II trials for adult and pediatric acute lymphocytic leukemia (287–289) and in one phase III trial (290). It was subsequently given FDA approval in 2017. Blinatumomab has also had positive initial results in the treatment of refractory non-Hodgkin lymphoma (291).

These developments have led to study of other targeted BITEs in multiple myeloma (292) hepatocellular carcinoma (293) and other solid tumors in conjunction with oncolytic virus and CAR-T therapies (172, 294). The expanding use of BITEs has been of particular interest in solid cancers that are relatively non-immunogenic, which may be poor candidates for checkpoint inhibition (295). As the most common neoantigen found in glioblastoma, EGFRvIII has been one of the first targets for BITEs therapy. Initial *in-vivo* work in murine glioma models showed high cure rates. Interestingly, after BITE therapy, immunosuppressive Tregs changed their behavior to attack EGFRvIII positive tumor cells via the granzyme-perforin pathway (296). In addition to targeting the EGFRvIII antigen, BITEs have also been applied to enhance the cytotoxic T cell response to cells expressing CD133+, a marker of tumor initiating cells in GBM (113). A preclinical study by Prasad et al. showed that BITEs targeting CD133 increased T cell ability to eradicate patient derived CD133+ GBM stem cells in orthotopic models of brain tumors (297). They showed that the BITEs specific to CD133 and CD3 were able to inhibit tumor progression in 4 out of the 5 mice, compared to control BITEs targeting prostate-specific membrane antigen (PSMA). They also confirmed the antitumor activity of the CD133 BITE in established tumors in orthotopic xenograft models by administering the bispecific antibodies on day 14 after the tumor implantation. In the group treated with PSMA BITEs, the tumor progressed between day 20 and 35, whereas the group treated with CD133 BITEs inhibited further tumor growth. Despite promising pre-clinical results, there are no current human trials for BITEs in glioblastoma (120).

## Conclusion

Glioblastoma is a highly malignant disease particularly resistant to the current armament of chemoradiation. As a result, the need for novel therapeutic strategies has been paramount. Over decades, research has made enormous strides in defining the pathogenesis of GBM and its relationship with its human host. A greater understanding of the immune function both systemically and within the tumor microenvironment has provided new therapeutic targets. In addition, in-depth knowledge of each patient's particular disease may allow for improved patient stratification to determine the best emerging therapy to use. The ongoing development of multiple immunotherapeutic strategies has ever-increasing potential to fundamentally change the way patients with glioblastoma are treated and, hopefully, make meaningful improvements in outcome.

## Author Contributions

JL provided the largest writing contribution to the manuscript and edited all sections of the manuscript. He was primarily responsible for assembling the manuscript and addressing comments by reviewers. VS performed literature and wrote multiple sections of the manuscript. She additionally assisted in creating figures and worked with medical arts to develop final figures. GD performed literature and wrote multiple sections of the manuscript and assisted in creating figures. AN provided additional literature review and writing of sections added to manuscript at time of review. EN provided extensive guidance, supervision, and editorial input to manuscript development.

### Conflict of Interest Statement

The authors refer to an ongoing clinical trial being performed by several of the authors. This trial receives funding from the Intramural Research Program at the National Institutes of Health and non-financial drug support from Bristol-Myers Squibb. Otherwise, the authors declare no potential conflicts of interest.
